# Contextual effects of social integration and disintegration on health status: evidence from South Korea

**DOI:** 10.1186/s12889-020-09077-7

**Published:** 2020-06-15

**Authors:** Eun-Bi Jo, Rang Hee Kwon, Minsoo Jung

**Affiliations:** 1 Department of Health Science, College of Natural Science, Dongduk Women’s University, 23-1 Wolgok-dong, Seongbuk-gu, Seoul, 136-714 South Korea; 2 Center for Community-Based Research, Dana-Farber Cancer Institute, Boston, MA USA

**Keywords:** Social integration, Social disintegration, Contextual effects, South Korea

## Abstract

**Background:**

Many studies have shown that various social integration variables represented by social capital are beneficial to communities, including collective health. However, the rapid decline in fertility rates and the breakup of familyism in developed countries require a new approach to social disintegration, but the literature is insufficient. Here, we explored the contextual effects of social integration and social disintegration on the health of individuals.

**Methods:**

The research data consist of merged datasets of 6909 respondents who were quota-sampled by approximately 30 people from 229 local governments in Korea. The individual-level independent variable is a social integration measure consisting of 26 questions in four areas. The community-level independent variables are five integral and aggregate variables extracted from 81 indicators. The dependent variable is self-rated health status. Potential confounders are gender, age, annual income, educational attainment, district type, and the number of beds in medical institutions per 1000 people.

**Results:**

The results showed that at the individual level, the higher the inclusive attitude of in- and out-of-networks, after adjusting for potential confounders, the less likely the respondent belongs to the unhealthy group (*p* < 0.001). At the community level, the higher the proportion of single-person households in a community after adjusting for potential confounders, the less likely the respondent belongs to the unhealthy group (*p* < 0.05). The effect size was 0.22.

**Conclusion:**

Thus far, social integration has been preferred, with the positive aspects of social capital being emphasized. However, this study shows that in some cases, social disintegration can instead positively influence an individual’s health. Therefore, further studies of the various conditions of social context effects on health are necessary.

## Background

Korean society has grown quantitatively over the past few decades, but economic inequality has deepened [1]. Since 2000, the total fertility rate and marriage rate have steadily declined, and the breakup of the family has been noticeable [2]. At the same time, gender equality appears to have worked towards dismantling patriarchal-based familyism [3]. As a result, demographic changes in Korean society appear to have crossed the Rubicon with regard to ultra-low fertility rates. However, little has been said about the social effects of ultra-low birth rates when coupled with a total fertility rate of less than 1.0. The collapse of the family is associated with a variety of phenomena and can have unintended consequences with regard to social integration and social disintegration [3]. In particular, little is known about whether such changes in the social structure can affect an individual’s health status or health inequalities. It is necessary to examine multiple facets of the concepts known as social integration and social disintegration to determine the associated health effects.

Health status is strongly associated with individual socioeconomic status indicators such as income, education, occupation and race [4, 5]. Recently, however, social integration has also been reported to have a significant contextual impact on health [6–10]. In particular, social capital, composed of social networks and social trust, helps to improve health to create potential information flows [11, 12]. However, more research is needed on the specific aspects of social capital. An abundance of social capital means that social members are more integrated due to high social trust [4]. This refers to the tendency of individuals to be more involved in social and political relationships, to embrace others, and to communicate. One result can be a positive impact on an individual’s health by fostering formal and informal social networks and strengthening reciprocity and mutual support [13].

However, there is still uncertainty with regard to the causal proposition that social integration is beneficial for health [14]. We seek to identify and add new contextual effect variables at the community level to this ambiguous association. Social integration based on social networks can have different effects depending on whether they are bonding or bridging connections [13]. Excessively closed or open networks can influence how individuals or groups are excluded from society [15]. Approaches based on social capital tend to reduce the macroscopic nature of this type of capital not being returned to the individual [16]. For example, if a community is materially wealthy and the crime rate is low, it can be judged that residents have strong bonds [17–19]. On the other hand, other communities may assume that if they have fewer children and a strong individualistic tendency, there will be less cohesion among those in the population [20, 21]. In each case, it is questionable as to whether different degrees of social integration can affect the health of the population. This study attempts to address this issue through a multilevel analysis.

## Methods

### Sample

Data were derived from a survey of respondents drawn from a nationally representative web-based sample of 6909 who participated in the Hankook Research Master Sample Panel. The respondents, adult men and women 20 years of age and older currently residing in South Korea, were proportionally selected by gender and age based on census data. The sample households were distributed according to the ratio of houses and apartments for each cluster (metropolis, city, and town). The final respondents were recruited by extracting 229 clusters from 15 provinces with a stratified cluster sampling method. The final response rate was 80.0%. Missing values of survey questions for key analytical variables were excluded using a pairwise method.

### Analytical framework

The contextual multilevel models employed in this study attempt to identify the effects of social integration on individual health status. We adopted a multilevel proposition, what is referred to here as a contextual multilevel approach, which shows the effect of the macro-level variable z on the micro-level variable y while controlling for the micro-level variable x [22]. The contextual multilevel approach permits a simultaneous examination of how individual- and group-level variables are related to individual-level health outcomes using community-level predictors. It accounts for the possibility of a residual correlation between individuals within groups and enables an examination of between-group variability and the factors associated with it [22, 23].

### Measures

The contextual effects model of this study is based on Blakely and Subramanian [24]. We used two types of indicators which show the integrative characteristics of community-level contextual effects. An aggregate indicator refers to the effect of a derived group-level variable on an individual-level outcome (e.g., mean educational attainment). An integral indicator refers to the effect of group-level variables that can apply to any situation involving lower-level units nested within higher-level units (e.g., gross regional domestic product; GRDP) [23, 25]. We employed the ratio of one-person households (ROPH) and the total fertility rate (TFR) as aggregate indicators and the number of four (murder, robbery, theft, violence) major crimes (NFMC), the number of beds in medical institutions per 1000 people (NBMI), and GRDP as integral indicators.

#### Dependent variable

The health outcome of this study is the self-rated health (SRH) status of residents. Respondents were asked to rate their own general health status on a ten-point Likert-type scale ranging from very good (10) to very bad (1) when responding to the question “How is your health in general?” The answers were eventually grouped into two categories. Respondents reporting 1 to 5 points for self-rated health status were coded as 1 and were considered as the low-SRH group, while those reporting 6 to 10 points self-rated health status were coded as 0 and were considered as the high-SRH group. In prospective studies, this general health question has been validated as a good predictor of morbidity and mortality, with a differential relationship between consecutive categorical ratings of SRH and the probability of mortality [26, 27].

#### Independent variables

Individual-level variables: Four dimensions of structured tools developed to measure social quality were used [28]: social communication, political participation, social participation as a form of neighborhoods and organizations, and social inclusion as a type of in- and out-of-network. Social participation (SP) refers to the degree of informal ties by which an individual is connected to his community [29–31], and social inclusion (SI) has been developed as a proxy for social efficacy [32]. All other variables were used in the Korean General Social Survey (KGSS; http://kgss.skku.edu/).

Community-level variables: Indicators were collected based on the European social quality (SQ) frame by Maesen and Walker [33]. This frame points out four conditions of a good society: socio-economic security, social cohesion, social inclusion and social empowerment, and proposes ninety-five sub-indicators. Based on this frame, we systematically collected usable indicators from 230 local governments in South Korea. The indicators were primarily collected from the central government statistics portal, which provides social indicators to local governments. The secondary sources were major public organizations followed by the Korea Public Information Disclosure System (https://www.open.go.kr). The portals used to collect indicators were the Korean Statistical Information Services (http://kosis.kr), the Local Administration Integrated Information System (http://www.laiis.go.kr), and the National Geographic Information Institute (http://nationalatlas.ngii.go.kr). We also used the expanded national database from the Ministry of Public Administration and Security, Ministry of Health and Welfare, Ministry of Culture, Sports and Tourism, the National Police Agency, the National Election Commission, the Health Insurance Review & Assessment Service, the Korea Education Development Institute, and the Korean Film Commission. Under the principles of conceptual suitability, clarity, reliability, consistency, changeability, and comparability, 81 indicators collected in total. Five indicators were selected using the Delphi method [7]. The variables representing social integration are the total fertility rate and GRDP, and the variables representing social disorganization are the number of four major crimes and the ratio of one-person households.

#### Standardization of Indicator

The indicators selected were transformed by the imputation of missing values, ESS (the European Social Survey) standardization, and GIS (Geographical Information System) transformation. First, the following were considered for missing values: 1) when there were no data from the year under examination, they were substituted with data from the previous year; 2) when there were no data from the previous year, they were substituted with data from the year before that; and 3) in other cases, the data were substituted with the average values for the province in which the region under examination was located. However, when the average values were simple sums, they were substituted with 1/n of the number of regions; additionally, when they were rate values, they were used in their entirety. Indicators were inputted after two-stage computations were calculated. First, we standardized the z-score of each indicator with the transformation formula of the European Social Survey in order to unify the units of measurement and form a normal distribution. The ESS methods enable us to minimize the bias of outlier observations as well as model complexity by transforming an indicator’s minimum and maximum values into 0 and 10, respectively, and its average into approximately 5 (http://essedunet.nsd.uib.no).

$$ t=\frac{5z\left(\max -\min \right)}{z\left(\min +\max \right)-2\min \ast \max }+5 $$

The medical resource factor consisted of two measures. The number of physicians is the aggregate number of certified physicians per 1000 people in a certain region. The number of general hospitals was computed by utilizing the GIS method so that we could take into consideration the availability of hospitals in neighboring regions to measure medical accessibility.

*The GIS-based number of general hospitals = the number of general hospitals in a certain region + (the neighboring region’s number of general hospitals / the number of neighboring regions) + (the secondary neighboring region’s number of general hospitals / the number of secondary neighboring regions).*

For example, suppose that there are three general hospitals in region *X*; at the same time, six general hospitals are in four neighboring regions and eleven general hospitals are in seven secondary neighboring regions. By substituting figures into the above formula, 3 + (6/4) + (11/7), we get a value of 6.07. This is the number of GIS-based hospitals in region *X*.

#### Potential confounders

The individual-level confounders were gender, age, annual income, educational attainment, and district type (metropolis, city, and town). The community-level confounders were the number of beds in medical institutions per 1000 people.

### Statistical analyses

The unconditional slope and conditional model of the multilevel model were devised for binary variables using the Bernoulli response and logit link respectively. Based on this model, the relationships between social integration/disorganization and low-SRH status at the individual and community levels were reviewed for statistical significance even after controlling for the relevant covariates.

#### Level 1 model (group-mean centering)

$$ {\displaystyle \begin{array}{l}\mathrm{Prob}\;\left( SRH=1\left|\beta \right.\right)=\varphi \\ {} Log\left[\varphi /\left(1-\varphi \right)\right]=\eta \end{array}} $$

$$ \eta ={\beta}_0+{\beta}_1\left(\mathrm{SC}\right)+{\beta}_2\left(\mathrm{PP}\right)+{\beta}_3\left(\mathrm{SP}\_\mathrm{N}\right)+{\beta}_4\left(\mathrm{SP}\_\mathrm{O}\right)+{\beta}_5\left(\mathrm{SI}\_\mathrm{in}\right)+{\beta}_6\left(\mathrm{SI}\_\mathrm{out}\right) $$

Note: SC: social communication; PP: political participation; SP_N: social participation (neighbors); SP_O: social participation (organizations); SI_in: social inclusion (in-network); SI_out: social inclusion (out-of-network).

#### Level 2 model (grand-mean centering)

$$ {\displaystyle \begin{array}{l}\beta ={\gamma}_{00}+{\gamma}_{01}\left(\mathrm{HOSPITAL}\right)+{\gamma}_{02}\left(\mathrm{GRDP}\right)+{\gamma}_{03}\left(\mathrm{TFR}\right)+{\gamma}_{04}\left(\mathrm{CRIME}\right)+{\gamma}_{05}\left(\mathrm{SINGLE}\right)+{\mu}_0\\ {}{\beta}_1={\gamma}_{10}+{\mu}_1\\ {}{\beta}_2={\gamma}_{20}+{\mu}_2\\ {}{\beta}_3={\gamma}_{30}+{\mu}_3\\ {}{\beta}_4={\gamma}_{40}+{\mu}_4\\ {}{\beta}_5={\gamma}_{50}+{\mu}_5\end{array}} $$

Note: Hospital: the number of beds of medical institution per 1000 people; GRDP: gross regional domestic product; TFR: the total fertility rate; CRIME: the number of four major crime; SINGLE: the ratio of one-person households.

In the logistic models, we calculated the intra-class correlation (ICC) using the formula σ^2^/(σ^2^ + 3.29), where σ^2^ is the area-level variance. The estimated size of the ICC based on the above model was 4.79%. The effect size was 0.22 (Nagelkerke value). The analysis procedure proceeded in the following order: descriptive statistics, factor and reliability analyses, and hierarchical generalized logistic regression. In the multilevel analysis, model 1 was the unconditional model, model 2 the unconditional slope model, and model 3 accounted for community-level and individual-level effects of social integration and disorganization. All analyses were conducted using HLM for Windows v.7.0.

### Ethics statement

This project was approved by the institutional review board of Dongduk Women’s University, Seoul, Korea (Sep 24, 2019; DDWU1909–01). Subjects provided written informed consent to participate in this study. No information that can publicly identify individual participants was collected during the data collection process.

## Results

### Descriptive statistics of the sample

The sample consisted of 48.4% men and 51.6% women (Table 1). At the individual level, 59.2% were aged from 30 to 49, 65.1% reported graduating from college, 49.3% earned from USD 10000 K to 39,999 K and 38.0% perceived their SRH status as low or very low. At the community level, the average numbers of hospital beds were 14.87 (±9.81) per 1000 and the average of GRDP was 7166 K per city districts. The average of total fertility rate was 1.16 (±0.262), the average numbers of the four major crimes was 2069.7 (±2128.5), and the average rate single household ratio was 30.8 (±5.2).

**Table 1:** Descriptive statistics for measures in a population-based analysis of the sample

Characteristics	Categories	Sample	Range		Mean ^a^
			Min	Max	
Individual-level (L1, *N* = 6909)					
Region, %	Town	2282			33.0%
	City	2362			34.2%
	Metropolis	2265			32.8%
Gender, %	Men	3341			48.4%
	Women	3568			51.6%
Age (yrs)	20–29	1113			16.1%
	30–39	1995			28.9%
	40–49	2094			30.3%
	50–59	1164			16.8%
	> = 60	543			7.9%
Educational attainment	Elementary school	25			0.4%
	Middle school	52			0.8%
	High school	1686			24.4%
	College	4499			65.1%
	Postgraduate	647			9.4%
Annual income (KRW)^b^	> 10,000 K	1312			19.0%
	10,000 ~ 19,999 K	815			11.8%
	20,000 ~ 29,999 K	1406			20.4%
	30,000 ~ 39,999 K	1181			17.1%
	40,000 ~ 49,999 K	740			10.7%
	50,000 ~ 59,999 K	566			8.2%
	60,000 ~ 69,999 K	331			4.8%
	70,000 ~ 79,999 K	232			3.4%
	80,000 ~ 89,999 K	135			2.0%
	90,000 ~ 99,999 K	81			1.2%
	> 100,000 K	110			1.6%
Self-rated health status	High	4285			62.0%
	Low	2624			38.0%
Community-level (L2, J = 229)^c^					
NBMI (2017)		228	0.02	67.7	14.87 (±9.81)
GRDP (2016)		223	287 K	59,981 K	7166 K (±9064 K)
TFR (2017)		227	0.646	2.099	1.16 (±0.262)
NFMC (2017)		226	8	13,471	2069.7 (±2128.5)
ROPH (2017)		229	18.5	46.0	30.8 (±5.2)

^a^Continuous variables are presented in mean (standard deviation) and categorical variables in percent (%)

^b^Equivalized annual income; KRW (Korean Won) 10,000 K = USD $8253.82 (Feb, 2020)

^c^NBMI (the number of beds of medical institution per 1000 people; GRDP (gross regional domestic product); TFR (the total fertility rate); NFMC (the number of four major crime); ROPH (the ratio of one-person households)

### Factor and reliability analyses

According to the results of the reliability analyses, the value of Cronbach α was satisfactory, with all factors being above 0.7 (Table 2). The first factor accounts for 57.24% of the variance of four items, referring to social communication. The second factor accounts for 46.59% of the variance of five items, referring to political participation. The third factor, social participation, was divided into neighborhood and organization. Social participation (neighborhood) accounted for 73.02% of the variance of the four items, and social participation (organization) accounted for 40.70% of the variance of seven items. The fourth factor, social inclusion, was divided into the in-network and out-of-network categories. Social inclusion (in-network) accounted for 49.11% of the variance of three items, and social inclusion (out-of-network) accounted for 45.57% of the variance of three items.

**Table 2:** Results of factor and reliability analyses on social integration

Factor	Item	Factor Loading						Reliability (Cronbach a)
		1	2	3	4	5	6	
Social Communication	Interfamily communication	0.839						0.736
	Communication between employees	0.832						
	Communication between neighbors	0.653						
	Intergenerational communication	0.625						
Political Participation	Present opinions to the government or the press		0.818					0.832
	Post any comments on the online bulletin board		0.811					
	Submit a petition		0.728					
	File a complaint with a civil servant		0.705					
	Participate in demonstrations or rally		0.683					
Social Participation (neighborhood)	Lending tools from neighbors			0.901				0.873
	Get help from neighbors			0.886				
	Have a casual conversation with neighbors			0.842				
	Visiting the neighbor unofficially			0.766				
Social Participation (organization)	Volunteer organization				0.735			0.821
	Community public organization (neighborhood associations, etc.)				0.715			
	Club (sports, leisure, culture, etc.)				0.698			
	Social gathering (reunion, hometown alumni, etc.)				0.682			
	Self-improvement groups (certification, etc.)				0.659			
	Socioeconomic organization (cooperative society, etc.)				0.654			
	Civic organization				0.594			
Social Inclusion (in-network)	The villagers get together well					0.887		0.841
	The villagers help their neighbors well					0.873		
	The villagers are reliable					0.847		
Social Inclusion (out-of-network)	Stranger						0.881	0.736
	Foreigners living in domestic						0.868	
	Neighborhood						0.617	
Eigen-value		2.019	4.095	2.535	5.749	1.893	1.294	
The Explained Variance (%)		57.24	46.59	73.02	40.70	49.11	45.57	

### Contextual effects on the low-SRH groups: multilevel analysis

The results of the HGLM analysis are shown in Table 3. At the individual level, the following results of model III were obtained: the higher the level of social communication, the less likely the respondent was to belong to a low-SRH group [aOR (adjusted odds ratio) = 0.611, 95% CI: 0.576–0.649]. However, the higher the level of political participation, the more likely the respondent was to belong to a low-SRH group (aOR = 1.107, 95% CI: 1.046–1.170). Regarding the factor of social participation, the results for neighbors and groups differed. The higher the level of social participation in organizations, the less likely the respondent was to belong to a low-SRH group (aOR = 0.853, 95% CI: 0.806–0.905). However, the higher the level of social participation with neighbors, the more likely the respondent was to belong to a low-SRH group (aOR = 1.132, 95% CI: 1.065–1.204). For the factor of social inclusion, the higher the level of social inclusion for both in-network (aOR = 0.797, 95% CI: 0.752–0.846) and out-of-network categories (aOR = 0.687, 95% CI: 0.650–0.726), the less likely the respondent was to belong to a low-SRH group. At the community level, the results of model III showed the following: the higher the level of NBMI, the more likely the respondent was to belong to a low-SRH group (aOR = 1.071, 95% CI: 1.001–1.145). The higher the level of GRDP, the less likely the respondent was to belong to a low-SRH group (aOR = 0.917, 95% CI: 0.839–0.993). However, the higher the level of NFMC, the more likely the respondent was to belong to a low-SRH group (aOR = 1.096, 95% CI: 1.038–1.240). The higher the level of ROPH, the less likely the respondent was to belong to a low-SRH group (aOR = 0.989, 95% CI: 0.882–0.990). The community-level variables were statistically significant except for the total fertility rate in the final conditional model).

**Table 3:** Multilevel model analyses affecting low-SRH status ^a^

Model parameter		Model I (unconditional)		Model II (unconditional slope)		Model III (conditional)	
		OR	95% CI	OR	95% CI	OR	95% CI
Level 1	Intercept, γ_00_	0.619	(0.586–0.654)	0.585	(0.551–0.620)	0.584	(0.551–0.618)
	Social Communication			0.612^e^	(0.576–0.650)	0.611^e^	(0.576–0.649)
	Political Participation			1.110^e^	(1.046–1.170)	1.107^e^	(1.046–1.170)
	Social Participation (neighbors)			1.132^e^	(1.065–1.203)	1.132^e^	(1.065–1.204)
	Social Participation (organizations)			0.855^e^	(0.807–0.905)	0.853^e^	(0.806–0.905)
	Social Inclusion (in-network)			0.798^e^	(0.752–0.846)	0.797^e^	(0.752–0.846)
	Social Inclusion (out-of-network)			0.689^e^	(0.652–0.727)	0.687^e^	(0.650–0.726)
Level 2^b^	NBMI					1.071^c^	(1.001–1.145)
	GRDP					0.917^c^	(0.839–0.993)
	TFR					0.985	(0.924–1.051)
	NFMC					1.096^c^	(1.038–1.240)
	ROPH					0.989^c^	(0.882–0.990)

^a^The gender, age, education, income, and residence type (city districts) of the respondents were adjusted

^b^NBMI (the number of beds of medical institution per 1000 people; GRDP (gross regional domestic product); TFR (the total fertility rate); NFMC (the number of four major crime); ROPH (the ratio of one-person households)

^c^: *p* < 0.05; ^d^: *p* < 0.01; ^e^e: *p* < 0.001

## Discussion

Today’s major developed countries are struggling with the concept of an ideal society beyond material abundance [34, 35]. Even if a country is economically rich, various social pathologies may arise due to relative inequalities among individuals [36–39]. In other words, a good society cannot be explained in terms of material things. In a similar vein, if GDP rises above a certain level, life expectancy rarely increases [40, 41]. Therefore, it is necessary to increase the healthy life span and reduce health inequalities among socioeconomic groups. This study examined what characteristics of society can contribute to reducing an individual’s low health status. Similar to what is known as Durkheim’s distinction between mechanical and organic solidarity, we explored contextual models that affect an individual’s health by collecting integral and aggregate variables that can reveal the unique effects of a community. This study uncovered two facts.

First, the effects of social integration and disintegration on the health of the population should be assessed in a multifaceted manner. In this study, where social integration was measured in a variety of conceptual constructs, political participation was associated with low health status. However, social participation showed contradictory results depending on whether the target of the relationship was a neighbor or an organization. Therefore, it is difficult to conclude that it is beneficial or harmful to health with only certain aspects of social integration or disintegration. However, an inclusive attitude toward social relations within and outside the individual’s network was consistently linked to a lack of good health. This is in line with previous studies that explored the benefits of social integration [41, 42]. Therefore, it is necessary to categorize various aspects of social integration and disintegration to classify the dimensions that are beneficial to the health of the population and those that are harmful.

Second, communities appear to be changing in favor of individual freedom over integration. According to the data in this study, an area with a large number of beds was 1.071 times more likely to be unhealthy. This appears to reflect the tendency of older people and patients to live in better medical facilities and to have access to better infrastructure. In addition, the higher the GRDP is, the lower the probability becomes of an unhealthy individual living in the area, by 0.917 times. Material abundance is a predictor that affects a person’s health in the community while also affecting the individual. However, while adjusting these two variables, the two types of social disintegration variables moved in the opposite direction. First, a high number of crimes, the classic indicator, was 1.096 times more likely to be worse with regard to the subjective health of individuals living in such an area. This is consistent with previous studies in that the rate of crime and/or the number of crimes, important variables for social disorganization, still have a negative effect on the health of individuals living in the community [43, 44]. However, it is interesting to note that the higher the proportion of single-person households in the community is, the less likely it is that the subjective health status of individuals living in the area is worse, by 0.989 times. This suggests, in contrast to previous studies, that social integration and social cohesion may at times not be beneficial to an individual’s health. Therefore, a further cross-national study is needed to determine whether our findings are valid only in the context of Korean society.

In countries such as Korea which have entered an era of ultra-low birth rates, the proportion of single-person households who do not voluntarily marry is high [45]. Existing social capital studies have tended to regard social networks as unconditionally positive [46, 47]. However, sometimes the pain, disadvantage, or fatigue from a relationship can outweigh the benefits [48, 49]. Therefore, in order for social integration to benefit an individual’s health, it must be based on healthy and beneficial social relationships. In addition, because there are many online communities in the internet age, relationships based on face-to-face contact may not be necessary [50–52]. Rather, people become more cautious in establishing social relationships, which can result in the dissolution of familyism.

This study has several limitations because it is an exploratory study. First, it cannot examine all variables that reveal social integration and disintegration. Therefore, more follow-up studies are necessary to understand this structural mechanism. Second, because the community was classified based on an administrative classification scheme, there is a limit when attempting to grasp the actual meaning of the individual qualitative relationship. Third, we used multilevel cross-sectional data to analyze only associations, not causation. Although the data used random-effects models to explain heterogeneity among regions, it was impossible totally to control qualitative heterogeneity among the 229 communities, which may have resulted in bias involving the estimated coefficients of the regression model. Further international comparative studies are needed to increase the internal and external validity of this study. Therefore, more sophisticated modeling is needed to discuss contextual effects that affect individual health. Nevertheless, this study is meaningful in that it attempts to grasp the integrative and/or disintegrative characteristics that affect the health of individuals in a rapidly changing society.

## Conclusion

In summary, many studies have shown that various social integration variables represented by social capital are beneficial to communities, including collective health. However, the rapid decline in fertility rates and the breakup of familyism in developed countries require a new approach to social disintegration, but the literature is insufficient. We explored the contextual effects of social integration and social disintegration on the health of individuals. The results showed that at the individual level, the higher the inclusive attitude of in- and out-of-networks, after adjusting for potential confounders, the less likely the respondent belongs to the unhealthy group. At the community level, the higher the proportion of single-person households in a community after adjusting for potential confounders, the less likely the respondent belongs to the unhealthy group. Thus far, social integration has been preferred, with the positive aspects of social capital being emphasized. However, this study shows that in some cases, social disintegration can instead positively influence an individual’s health. Therefore, further studies of the various conditions of social context effects on health are necessary.

## Data Availability

Data sharing: Participant level data are available from the corresponding author.

## Abbreviations

SRH: Self-Rated Health
GIS: Geographical Information System
GRDP: Gross Regional Domestic Product
ROPH: Ratio of one-person households
TFR: Total Fertility Rate
NFMC: Number of four (murder, robbery, theft, violence) major crime
NBMI: Number of beds of medical institution
ESS: European Social Survey
ICC: Intra-class correlation

## References

[1] Son M, An SJ, Kim YJ (2017). Trends of social inequalities in the specific causes of infant mortality in a nationwide birth cohort in Korea, 1995-2009. J Korean Med Sci.

[2] Chung S (2010). Causal model of low fertility determinants in Korea. J Soc Sci.

[3] Hasunuma L, Shin K (2019). #MeToo in Japan and South Korea: #WeToo, #WithYou. J Women Politics Pol.

[4] Campos-Matos I, Subramanian SV, Kawachi I (2016). The ‘dark side’ of social capital: trust and self-rated health in European countries. Eur J Pub Health.

[5] Williams DR (1999). Race, socioeconomic status, and health: the added effects of racism and discrimination. Ann N Y Acad Sci.

[6] Subramanian SV, Kim DJ, Kawachi I (2002). Social trust and self-rated health in US communities: a multilevel analysis. J Urban Health.

[7] Poortinga W (2006). Social capital: An individual or collective resource for health?. Soc Sci Med.

[8] Rostila M (2017). Social capital and health in European welfare regimes: a multilevel approach. J Eur Soc Policy.

[9] Jen MH, Sund ER, Johnston R, Jones K (2010). Trustful societies, trustful individuals, and health: An analysis of self-rated health and social trust using the world value survey. Health Place.

[10] Kawachi I (2017). Trust and population health. In: Uslaner EM, Kawachi I (Eds.). The Oxford handbook of social and political trust (pp. 1–35).

[11] Putnam RD (2007). E pluribus Unum: diversity and community in the twenty-first century. The 2006 Johan Skytte prize lecture. Scand Political Stud.

[12] Putnam RD (1993). Making democracy work. Civic Traditions in modern Italy.

[13] Rapp C, Huijts T, Eikemo TA, Stathopoulou T (2018). Social integration and self-reported health: differences between immigrants and natives in Greece. Eur J Pub Health.

[14] Giordano GN, Lindstrom M (2016). Trust and health: testing the reverse causality hypothesis. J Epidemiol Community Health.

[15] Waytz A, Epley N (2012). Social connection enables dehumanization. J Experiment Soc Psychol.

[16] Oshio T (2016). The association between individual-level social capital and health: cross-sectional, prospective cohort and fixed-effects models. J Epidemiol Community Health.

[17] Akçomak İS, Ter Weel B (2012). The impact of social capital on crime: evidence from the Netherlands. Reg Sci Urban Econ.

[18] Saegert S, Winkel G (2004). Crime, social capital, and community participation. Am J Community Psychol.

[19] Takagi D, Ikeda KI, Kobayashi T, Harihara M, Kawachi I (2016). The impact of crime on social ties and civic participation. J Community App Soc Psychol.

[20] Bühler C, Fratczak E (2007). Learning from others and receiving support: the impact of personal networks on fertility intentions in Poland. Eur Soc.

[21] Philipov D, Spéder Z, Billari FC (2006). Soon, later, or ever? The impact of anomie and social capital on fertility intentions in Bulgaria and Hungary. Pop Stud.

[22] Snijders TAB, Bosker RJ (1999). Multi-level analysis: An introduction to basic and advanced multi-level modeling.

[23] Diez-Roux AV (2002). A glossary for multilevel analysis. J Epidemiol Community Health.

[24] Blakely T, Subramanian SV. Multilevel studies. In: Oakes J, Turner J, editors. Methods for social epidemiology (pp. 316-40), vol. 42. San Francisco: Jossey-Bass; 2006.

[25] Diez-Roux AV (2003). The examination of neighborhood effects on health: conceptual and methodological issues related to the presence of multiple levels of organization. In LF Berkman, I Kawachi (Eds.), neighborhoods and health (pp. 45–64).

[26] DeSalvo KB, Bloser N, Reynolds K, He J, Muntaner P (2005). Mortality prediction with a single general self-rated health question: a meta-analysis. J Gen Int Med.

[27] Idler EL, Benyamini Y (1997). Self-rated health and mortality: a review of twenty-seven community studies. J Health Soc Behav.

[28] Koo HR, Jung M (2013). Exploring regional disparities: the social quality approach. Korean Local Admin Rev.

[29] Ross CE, Jang SJ (2000). Neighborhood disorder, fear, and mistrust: the buffering role of social ties with neighbors. Am J Community Psychol.

[30] Putnam R (2000). Bowling alone: the collapse and revival of American community.

[31] Jung M, Viswanath K (2013). Does community capacity influence self-rated health? Multilevel contextual effects in Seoul. Korea Soc Sci Med.

[32] Sampson RJ, Raudenbush SW, Earls F (1997). Neighborhoods and violent crime: a multilevel study of collective efficacy. Science..

[33] Maesen LJG, van der Walker AC (2005). Indicators of social quality: outcomes of the European scientific network. Eur J Social Qual.

[34] Sachs JD (2012). From millennium development goals to sustainable development goals. Lancet..

[35] Xiao Y, Norris CB, Lenzen M, Norris G, Murray J (2017). How social footprints of nations can assist in achieving the sustainable development goals. Ecological Econ.

[36] Choe J (2008). Income inequality and crime in the United States. Econ Lett.

[37] Khang YH, Cho HJ (2006). Socioeconomic inequality in cigarette smoking: trends by gender, age, and socioeconomic position in South Korea, 1989–2003. Prev Med.

[38] Odgers CL (2015). Income inequality and the developing child: is it all relative?. Am Psychol.

[39] Wildman J (2001). The impact of income inequality on individual and societal health: absolute income, relative income and statistical artefacts. Health Econ.

[40] Linden M, Ray D (2017). Aggregation bias-correcting approach to the health–income relationship: life expectancy and GDP per capita in 148 countries, 1970–2010. Econ Model.

[41] Swift R (2011). The relationship between health and GDP in OECD countries in the very long run. Health Econ.

[42] Seeman TE (1996). Social ties and health: the benefits of social integration. Ann Epidemiol.

[43] Kawachi I, Kennedy BP, Wilkinson RG (1999). Crime: Social disorganization and relative deprivation. Soc Sci Med.

[44] Sampson RJ (2003). Neighborhood-level context and health: lessons from sociology.

[45] Jones GW (2007). Delayed marriage and very low fertility in Pacific Asia. Pop Development Rev.

[46] Hawe P, Shiell A (2000). Social capital and health promotion: a review. Soc Sci Med.

[47] Yip W, Subramanian SV, Mitchell AD, Lee DTS, Wang J, Kawachi I (2007). Does social capital enhance health and well-being? Evidence from rural China. Soc Sci Med.

[48] Allik J, Realo A (2004). Individualism-collectivism and social capital. J Cross-Cultural Psychol.

[49] Kagitcibasi C (1997). Individualism and collectivism. Handbook Cross-Cultural Psychol.

[50] Best SJ, Krueger BS (2006). Online interactions and social capital: distinguishing between new and existing ties. Soc Sci Computer Rev.

[51] Ellison NB, Steinfield C, Lampe C (2007). The benefits of Facebook “friends:” social capital and college students’ use of online social network sites. J Computer-Mediated Communic.

[52] Kobayashi T, Ikeda K, Miyata K (2006). Social capital online: collective use of the internet and reciprocity as lubricants of democracy. Info Community Soc.

